# Application of targeted next-generation sequencing for pathogens diagnosis and drug resistance prediction in bronchoalveolar lavage fluid of pulmonary infections

**DOI:** 10.3389/fcimb.2025.1590881

**Published:** 2025-06-09

**Authors:** Qiugui Xu, Qiumei Chen, Wen Qiu, Lili Liu, Wan Zeng, Jinling Chen, Yangyang Li, Zhen Guo, Ling Rong, Bigui Chen, Jinxiu Yao, Liye Yang

**Affiliations:** ^1^ Clinical Laboratory, People’s Hospital of Yangjiang, Yangjiang, Guangdong, China; ^2^ Department of Radiology, People’s Hospital of Yangjiang, Yangjiang, Guangdong, China; ^3^ Precision Medical Lab Center, People’s Hospital of Yangjiang, Yangjiang, Guangdong, China; ^4^ Clinical Laboratory, Chest Hospital of Zhengzhou University, Zhengzhou, Henan, China; ^5^ Department of Respiratory and Critical Care Medicine, People’s Hospital of Yangjiang, Yangjiang, Guangdong, China

**Keywords:** targeted next-generation sequencing (TNGS), conventional microbiological tests (CMTs), pulmonary infection, bronchoalveolar lavage fluid (BALF), pathogens

## Abstract

**Background:**

Recently, targeted next-generation sequencing (tNGS) have been extensively utilized for the identification of pathogens in pulmonary infections, there have been some studies systematically evaluating differences in the efficacy of tNGS and conventional microbiological tests (CMTs) in bronchoalveolar lavage fluid (BALF) specimens.

**Methods:**

A retrospective analysis was conducted on 203 patients with pulmonary infections treated in one tertiary hospital from July 2023 to February 2024. BALF specimens underwent parallel testing via tNGS and CMTs. Pathogen detection consistency, the drug resistance genes concordance with phenotypic drug sensitivity, and clinical impact of tNGS-guided therapy adjustments were analyzed. Furthermore, two patients with complex infections were selected for tNGS microbiological surveillance to evaluate the efficacy of monitoring severe pneumonia.

**Results:**

This study included 205 confirmed infectious BALF specimens (two patients were tested twice). tNGS identified 56 putative pathogens, compared to 20 by CMTs, with a significantly higher positive rate (99.5% vs. 35.6%, *P*<0.0001). The detection of pathogenic microorganisms using tNGS showed a high concordance rate with the results of CMTs. tNGS-guided therapy adjustments occurred in 17.2% (35/203) of patients. Resistance gene predictions aligned with the drug sensitivity results in 40% (6/15) of carbapenem-resistant organisms (CROs) and 80% (4/5) of methicillin-resistant Staphylococcus aureus (MRSA) cases. Additionally, for monitored two patients with severe pneumonia, the tNGS results were consistent with the culture and imaging test results during treatment.

**Conclusions:**

The application of tNGS highlights its promise and significance in identifying potential pathogens, predicting drug resistance, and providing guidance for anti-infection therapies for severe pneumonia. It can be at least a complementary approach to CMTs reporting.

## 1 Introduction

Various pulmonary infections continue pose a serious threat to human life and health (GBD 2021 Diseases and Injuries Collaborators, 2024). In recent years, the diagnosis and treatment of pulmonary infections face many challenges due to the increasing drug resistance of common pathogens causing pneumonia, the rising incidence of tuberculosis coupled with a slowing prevention and treatment process, and the ongoing mutation and prevalence of viruses leading to increased susceptibility to fungi (Antimicrobial Resistance Collaborators, 2022; Motta et al., 2024; Chen et al., 2022). The timely and accurate pathogen diagnosis plays a crucial role in the clinical diagnosis and treatment of pulmonary infections (Caliendo et al., 2013; Cilloniz et al., 2016). Currently, the identification of pathogens mainly relies on conventional microbiological tests (CMTs), including culture, microscopic smears, polymerase chain reactions (PCR) and serologic testing. However, CMTs have certain limitations, such as time-consuming, complex operations, and low detection rates, especially for uncommon pathogens and difficult-to-culture organisms, making them inadequate to meet clinical needs (Rajapaksha et al., 2019; Zhao et al., 2024). With the continuous development of sequencing technology, the emergence of metagenomic next-generation sequencing (mNGS) has brought significant advancements in the field of infectious disease diagnosis, particularly in the area of pulmonary infections (Hu et al., 2024). Compared with CMTs, mNGS can rapidly, accurately, and comprehensively identify pathogens, making it a powerful tool for the detection, identification, and analyses of pathogens (Li et al., 2020; Diao et al., 2023; Huang et al., 2024). However, mNGS technology also has drawbacks, such as interference of host sequences, inability to simultaneously conduct DNA/RNA dual-processing detection, and high costs, which limit its widespread application in clinical settings. Targeted next-generation sequencing (tNGS) technology combines ultra-multiplex PCR with high-throughput sequencing, enabling targeted detection of multiple pathogens (ranging from dozens to hundreds of common microbial pathogens) and resistance genes, which provides options to overcome the current bottlenecks of mNGS technology (Wei et al., 2024; Li et al., 2022; Sun et al., 2024).

Bronchoalveolar lavage fluid (BALF) and sputum are both commonly used sample types from the lower respiratory tract. BALF, due to directly obtained from the lesion site, can reduce contamination from oral flora and improve the isolation rate of pathogens. Therefore, BALF specimens can enhance the efficacy of pathogen detection. However, there are few studies on summarizing the performance of tNGS for pathogen identification in BALF specimens. The aim of this study was 2-fold. First, we systematically evaluated the differences in pathogenic diagnostic performance between tNGS and CMTs in BALF specimens, by collecting 205 clinical BALF specimens. Second, we evaluated the use of tNGS in the predictive effectiveness for carbapenem-resistant organisms (CROs) and methicillin-resistant Staphylococcus aureus (MRSA).

## 2 Materials and methods

### 2.1 Patients and samples

**Figure 1:**
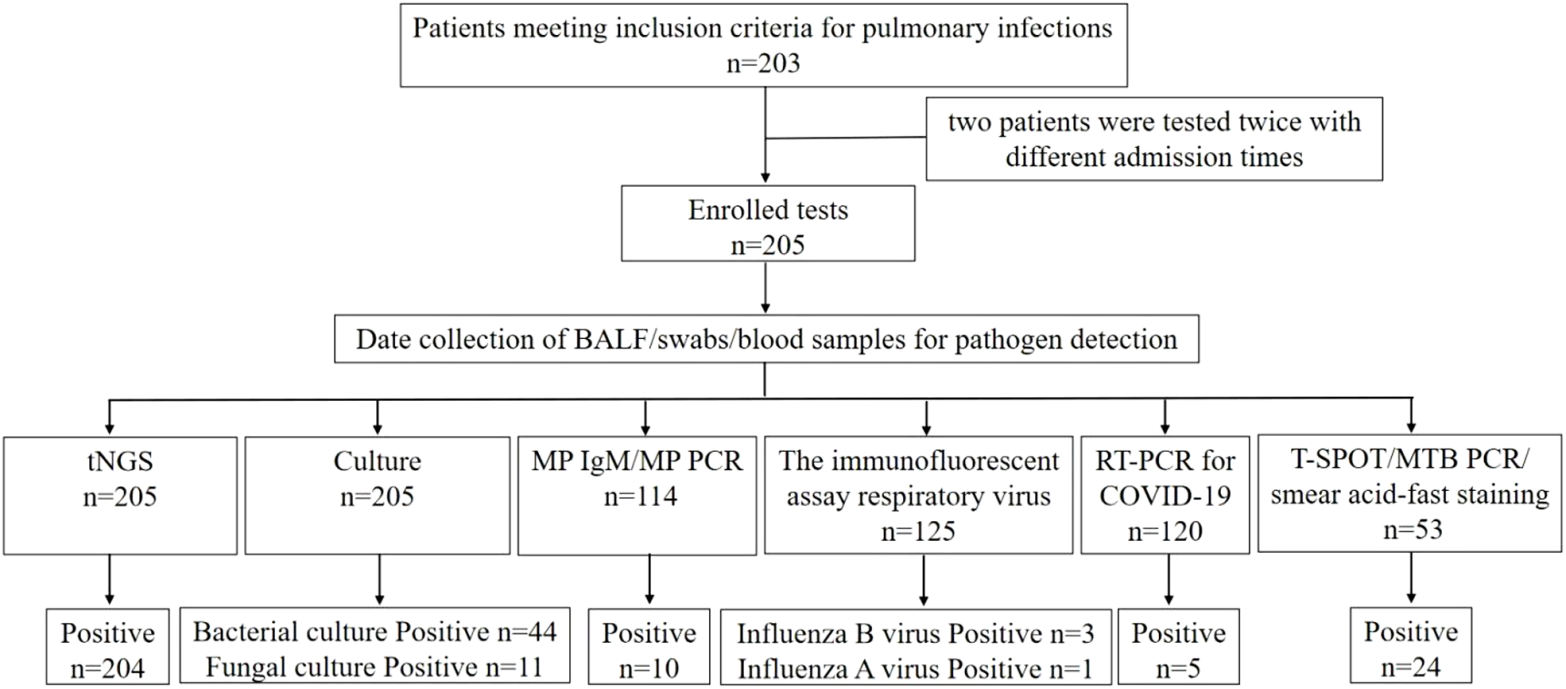
Flowchart of screening of patients with pulmonary infections and detection results of difference methods.

This retrospective case series was conducted on patients with pulmonary infections admitted to the Respiratory and Critical Care Medicine Department of People’s Hospital of Yangjiang between July 2023 and February 2024. The study protocol was approved by the Ethics Review Committee of People’s Hospital of Yangjiang (No:20230003). A total of 203 patients were included for analysis, and their clinical data were collected (**Figure 1**). Inclusion criteria were as follows: (1) Patients diagnosed with pulmonary infections based on clinical characteristics, imaging findings, inflammatory markers and pathogen detection; (2) Patients on whom tNGS/CMTs were performed; (3) BALF specimens; (4) Patients with complete clinical data. Exclusion criteria: (1) BALF samples or the testing process failed to pass the quality control for tNGS; (2) insufficient case data.

### 2.2 Sample collection

#### 2.2.1 BALF collection

A skilled bronchoscopist performs bronchoscopy to obtain BALF specimens. After nebulized anesthesia with 5ml of 2% lidocaine, the bronchoscope is inserted through the nostril. Based on the patient’s pulmonary CT scan results, the lesion site is selected for bronchoscopic alveolar lavage. If the pulmonary imaging shows diffuse changes, the middle lobe of the right lung or the lingula of the left upper lobe is choosed for lavage. After the fiberoptic bronchoscope reaches the target lung segment and becomes wedged, 20 ml of sterile saline is injected through the operating channel of the bronchoscope each time, with a total injection volume ranging from 60 to 120 ml.

After injecting the sterile saline, negative pressure (100 mmHg) is immediately applied for aspiration, with a total recovery rate of no less than 40%. The recovered lavage fluid is stored in a sterile container. Two tubes of lavage fluid (≥10 ml each) were collected, one for routine bacterial and fungal culture, and the other for tNGS.

#### 2.2.2 Routine specimen collection

Nasopharyngeal swabs and venous blood samples are collected by trained nurses. Nasopharyngeal swabs are used for viral antigen testing (including influenza A/B virus, respiratory syncytial virus, adenovirus, parainfluenza virus type 1/2/3), as well as for quantitative reverse transcription PCR (RT-qPCR) analysis for COVID-19. Venous blood is used for routine blood tests, procalcitonin (PCT), C-reactive protein (CRP), *Mycoplasma pneumoniae* (MP) IgM antibody, and *Chlamydia pneumoniae* (CP) IgM antibody testing. If necessary, T-cell spot test for *Mycobacterium tuberculosis* (MTB) infection (T-SPOT.TB), MTB PCR, and MP PCR are performed.

### 2.3 tNGS assay

After specimen collection, they are sent to KingMed Co., Ltd. (Guangzhou, China) for tNGS testing.

#### 2.3.1 Sample preparation and nucleic acid extraction

Viscous samples were liquefied, and the precipitate was collected. The precipitates were transferred into a grinding tube, followed by the addition of lysis buffer. Then, the samples were lysed using a shock crusher. Subsequently, nucleic acid was automatically extracted on the KingFisher flex automated extraction Instrument and with the Magnetic Pathogen DNA/RNA Kit (Magen Biotech Co., Ltd, Guangzhou, China).

#### 2.3.2 Library preparation and sequencing analysis

cDNA synthesis and library preparation were performed using the respiratory multi-pathogen targeted sequencing kit (KingCreate Biotechnology Co., Ltd., Guangzhou, China). After cDNA synthesis, the target region PCR products were purified using magnetic beads, then the libraries were amplified with the collected products. The quality control (QC) of DNA libraries was carried out using a Qubit Fluorometer (Thermo Fisher Scientific, MA, USA) to assess DNA concentrations. Sequencing was performed using a KM MiniSeqDx-CN Gene Sequencer and the Sequencing Reaction Universal Kit (KingCreate Biotechnology Co., Ltd., Guangzhou, China). The analysis report was automatically generated using the “Targeted Pathogen Detection (tNGS) Data Analysis Management System” Version 2.5.0 (KingCreate, Guangzhou, China). The bioinformatics analysis processes were as follows: (1) The fastp program was used to conduct basic quality control and filter the data; (2) The filtered reads were matched against the tNGS-specific database using Bowtie 2 software. Data quality requirements were included: Q30 ≥ 75%, minimum raw reads ≥ 50k, and internal reference gene amplification reads ≥ 200. The tNGS assay is a targeted detection of 198 pathogens and 15 types of drug resistance genes (**Supplementary Table S**).

#### 2.3.3 Result interpretation

The report interpretation team for tNGS includes microbiology laboratory technicians/physicians and clinical physicians. In tNGS reports, pathogenic, conditionally pathogenic, and colonizing/commensal microorganisms are classified based on the pathogenicity of organisms within specific sample types. The determination of whether a microorganism is an actual pathogen requires comprehensive assessment of clinical context. Pathogenic microorganisms are considered pathogens. Conditionally pathogenic microorganisms require comprehensive evaluation of immune status, host factors, laboratory parameters, imaging features, medication history, treatment response, and traditional microbiological test results to determine their role as pathogens. Colonizing or commensal microorganisms are generally not regarded as pathogens; however, in cases of immunosuppression, consistency with other microbiological findings, or alignment with clinical diagnosis, they may be considered as one of the pathogen indicators (Society of Clinical Microbiology of China International Exchange and Promotion Association for Medical and Healthcare, 2024; The Laboratory Medicine Society, China Association of Medical Equipment, 2025).

### 2.4 Process of CMTs

#### 2.4.1 Microbial culture

BALF samples were cultured on blood agar, chocolate agar, MacConkey agar and Sabouraud agar (Antu Biological Co., Ltd., Zhengzhou, China) at 35°C. Sabouraud agar (Antu Biological Co., Ltd., Zhengzhou, China) incubated at 35°C was used to isolate fungi. Bacteria were identified by the Autof ms600 automated microbial mass spectrometer. The drug sensitivity testing was performed by Vitek2 automated system (BioMérieux, Marcy-l’Etoile, France).

#### 2.4.2 Respiratory virus Screening

The immunofluorescent assay Respiratory Virus Kit (Hybrids, Shanghai, China) to simultaneously tested antigens of respiratory virus (including influenza A/B virus, respiratory syncytial virus, adenovirus, parainfluenza virus type 1/2/3). The Nasopharyngeal swabs were gathered in a specialized tube containing physiological saline. The specialized tube was added into 5 ml PBS solution to remove the mucus, which obtained cell suspension. The cell suspension was added to a specific slide, and a seven-item respiratory virus fluorescent screening reagent was added, which incubated at 37°C for 15–30 minutes. Subsequently, the slide was washed with PBS solution, and mounting medium was added. The stained and mounted sample slide was observed using a fluorescence microscope (Nikon, Tokyo, Japan) at 100x magnification.

#### 2.4.3 RT-qPCR procedure for COVID-19

The COVID-19 was analyzed using the COVID-19 Real-time PCR Kit (Biogerm, Shanghai, China). The oral pharyngeal swabs were gathered and maintained at 2–8 °C. The RNA was isolated within 48 h using a kit designed for nucleic acid extraction or purification (Biogerm, Shanghai, China). Amplification and quantitation were performed in the BIO-RAD CFX96 Real-Time PCR Systems (BIO-RAD, California, USA) according to the manufacturers’ instructions. A cycle threshold (CT) value ≤ 35 was considered positive.

#### 2.4.4 Serologic testing

The *Chlamydia pneumoniae* and *Mycoplasma pneumoniae* underwent an analysis using the CP IgM & MP IgM Kit (Livzon, Zhuhai, China) according to the manufacturers’ instructions.

### 2.5 Statistical analysis

GraphPad Prism 8 software (GraphPad Software, Inc.) was used for the data analysis. Continuous variables conforming to a normal distribution were expressed as mean ± standard deviation. Those with a non-normal distribution were presented as the median with the interquartile range [M (P25, P75)]. The frequencies and percentages [N (%)] were utilised for categorical variables, and comparisons between groups were conducted using χ2 test as appropriate. A two-tailed value of *P* of <0.05 was regarded as statistically significant.

## 3 Results

### 3.1 Demographic characteristics

A total of 205 BALF specimens from 203 patients were initially enrolled in this study according to the inclusion and exclusion criteria. Among them, two patients were tested twice with different admission times. The patient demographic information, admission symptoms, underlying diseases and laboratory findings are presented in **Table 1**.

**Table 1:** Patient demographics.

Characteristic	N (%)
Age, M (P25, P75)	65 (45, 74)
Gender, N (%)	
Male	130 (64.0)
Female	73 (36.0)
Admission symptoms, N (%)	
Cough	174 (85.7)
Expectoration	140 (69.0)
Fever	131 (64.5)
Dyspnea	66 (32.5)
Hemoptysis	16 (7.9)
Chest pain	15 (7.4)
Other symptoms	56 (27.6)
Underlying diseases, N (%)	
Hypertension	38 (18.7)
Tumors	37 (18.2)
Diabetes	26 (12.8)
Chronic respiratory diseases	21 (10.3)
Cerebrovascular diseases	19 (9.4)
Chronic cardiac diseases	18 (8.9)
Laboratory findings	
WBC (10^9^ /L), M (P25, P75)	8.7 (6.12, 11.45)
Proportion of neutrophils (%), M (P25, P75)	74.7 (66.7, 83.6)
CRP	39.15 (14.39, 109.6)
PCT	0.1 (0.1, 1.2)

WBC, White blood cell; PCT, procalcitonin; CRP, C-reactive protein.

### 3.2 Pathogens identification by tNGS and CMTs

**Figure 2:**
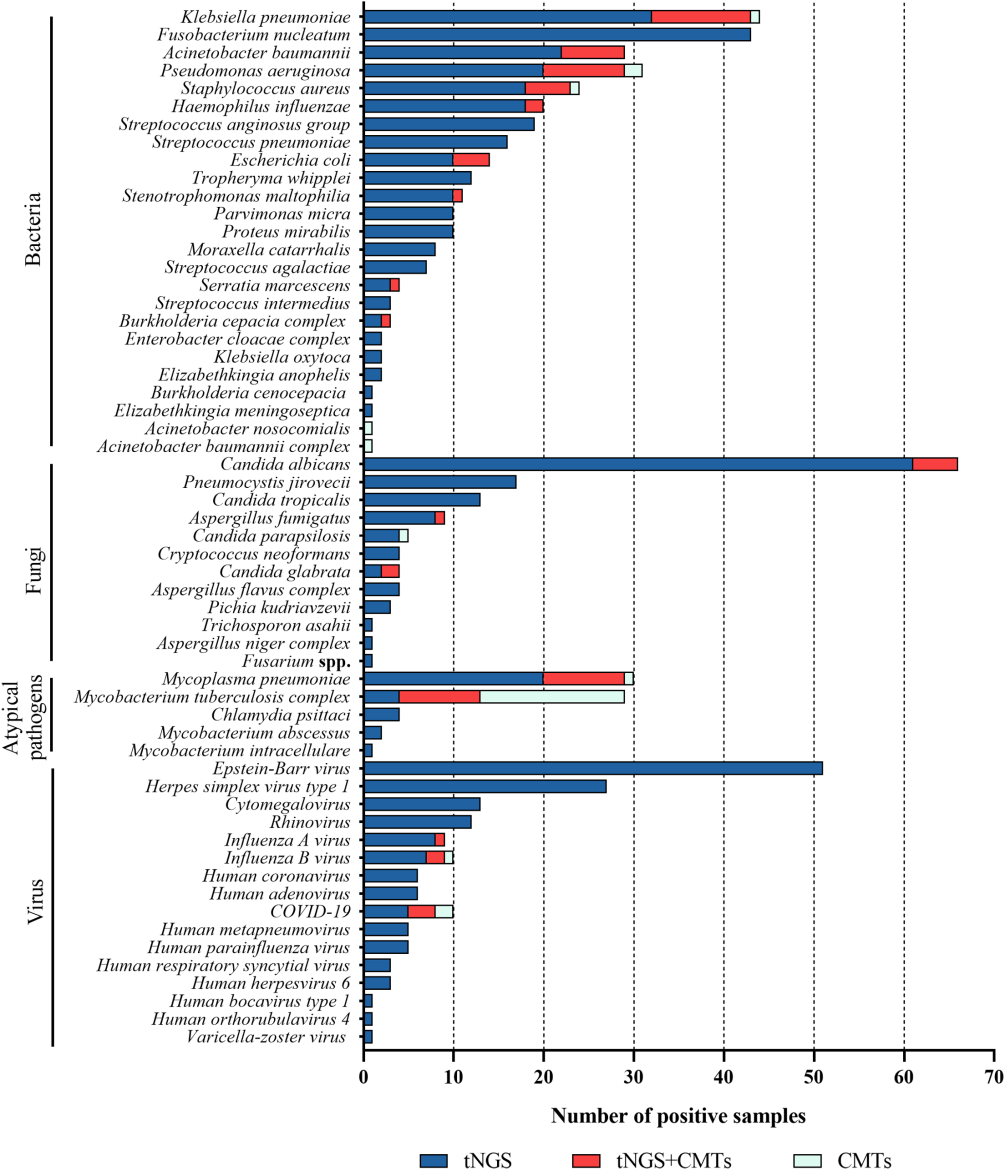
Distribution of potential pathogens in the study cohort and the pathogen detection results of tNGS and CMTs, respectively.

**Figure 3:**
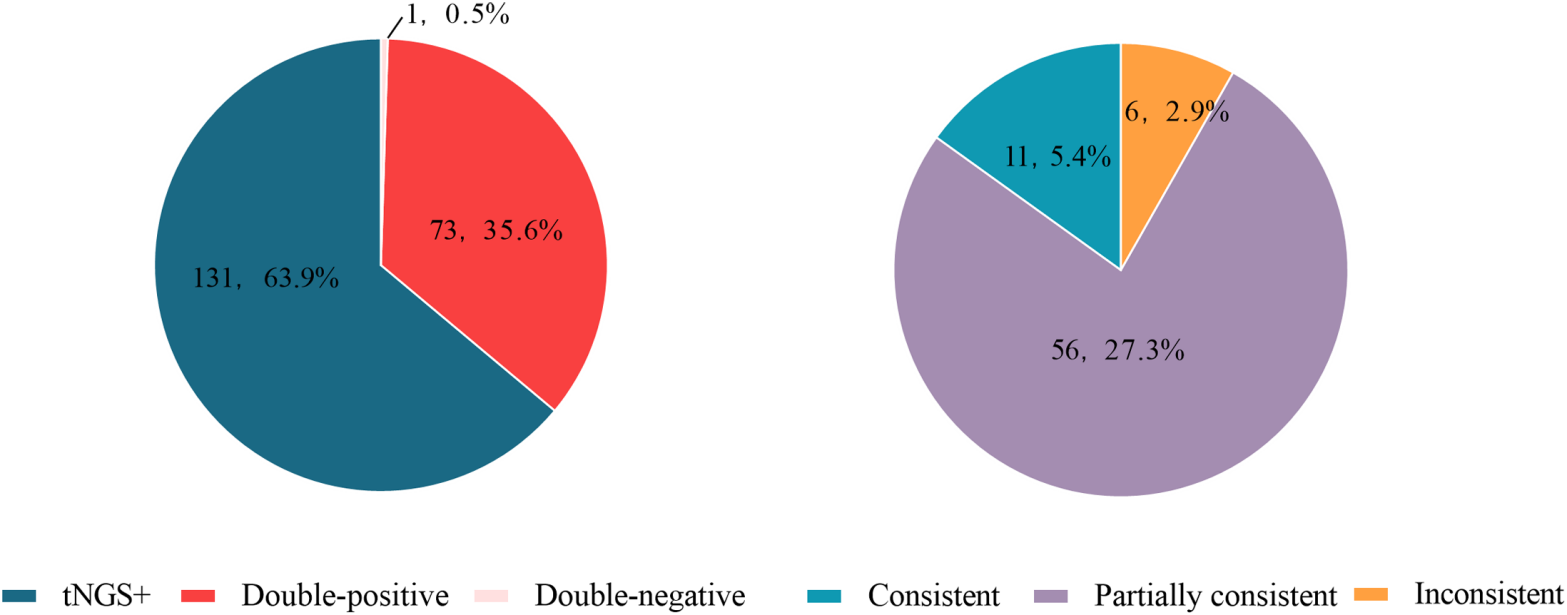
Consistency between tNGS and CMTs for pathogen detection.

Among the 205 enrolled tests, 56 types of pathogens were detected by tNGS, whereas only 20 types were detected by CMTs (**Figure 2**). The positive rate (number of positive results/number of cases tested) of tNGS was 99.5% (204/205), significantly higher than that of CMTs at 35.6% (73/205) (*P*<0.0001). There were 73 tests (34.6%) that detected positive by both tNGS and CMTs. Among these, 11 tests (5.4%) had completely consistent pathogen detection results between tNGS and CMTs, 56 tests (27.3%) had partially consistent results, and 6 tests (2.9%) had completely inconsistent results. There was 1 test (0.5%) that detected negative by both methods. Notably, 131 tests (63.9%) detected positive only by tNGS, and no test detected positive only by CMTs (**Figure 3**).

### 3.3 Bacterial pathogens identification by tNGS and CMTs

**Figure 4:**
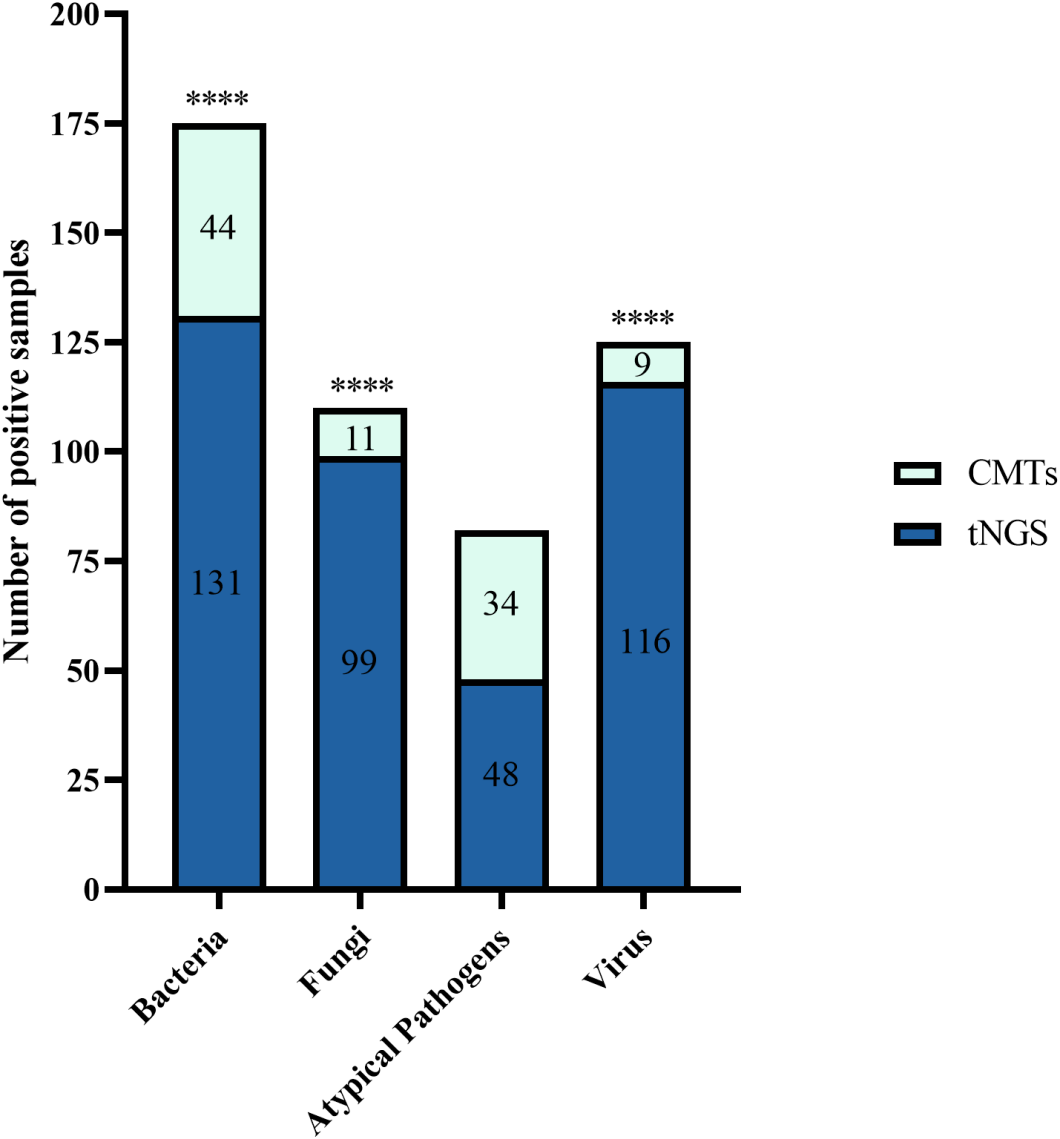
The bar plot shows the total number of positive samples for each category. ****P < 0.0001.

**Figure 5:**
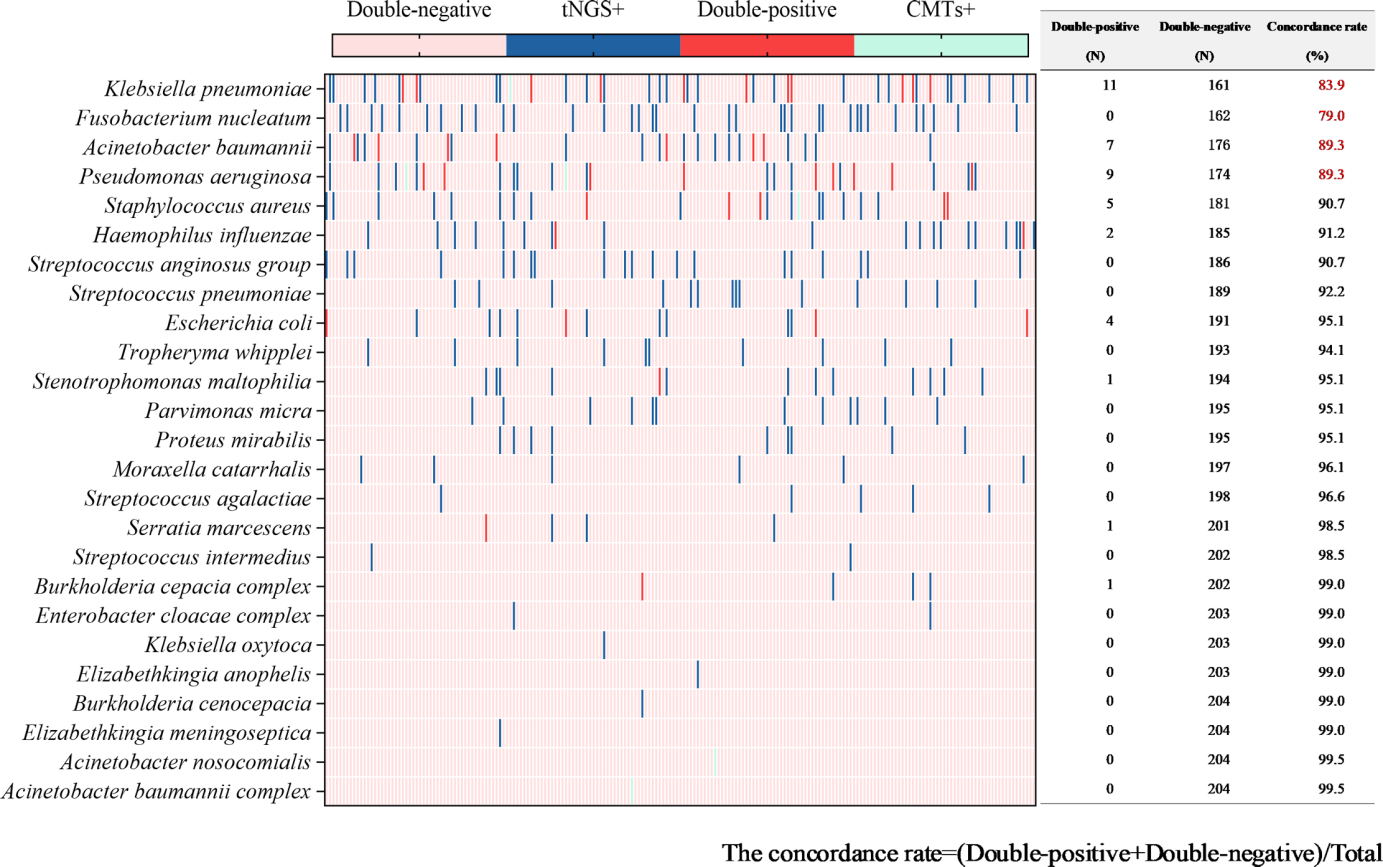
Concordance analysis between tNGS and CMTs assays in bacterial pathogens. Heat map displaying the result of bacterial pathogens detected by tNGS and CMTs.

In 205 tests of BALF, bacterial pathogens were detected in 131 tests (63.9%) via tNGS, totaling 313 strains. *Klebsiella pneumoniae* and *Fusobacterium nucleatum* were the most prevalent (43 tests each), followed by *Pseudomonas aeruginosa* and *Acinetobacter baumannii* (29 tests each), *Staphylococcus aureus* (23 tests), *Haemophilus influenzae* (20 tests), *Streptococcus anginosus group* (19 tests), and *Streptococcus pneumoniae* (16 tests). Bacterial pathogens were detected in 44 tests (21.5%) by CMTs (**Figure 4**). Among them, *Klebsiella pneumoniae* was the most frequently detected bacterium (12 tests), followed by *Pseudomonas aeruginosa* (11 tests) and *Acinetobacter baumannii* (7 tests). When comparing the two methods for detecting bacteria, 42 tests (20.5%) were positive by both methods, 72 tests (35.1%) were negative by both methods, 89 tests (43.4%) were positive only by tNGS, and 2 tests (1.0%) were positive only by CMTs. The concordance rates for various detected bacteria are shown in **Figure 5**. The concordance rates for *Klebsiella pneumoniae*, *Fusobacterium nucleatum*, *Pseudomonas aeruginosa*, and *Acinetobacter baumannii* are below 90%, which is due to the high positive rate of these four bacteria solely by tNGS. Then, we evaluated the detection performance of tNGS. Using bacterial culture as the gold standard, tNGS demonstrated sensitivity of 95.12% and specificity of 44.72% (**Supplementary Figure 1A**). Among these, the three tests where the bacteria detected by tNGS and culture were completely inconsistent were excluded from the statistical analysis.

### 3.4 Fungal pathogens identification by tNGS and CMTs

**Figure 6:**
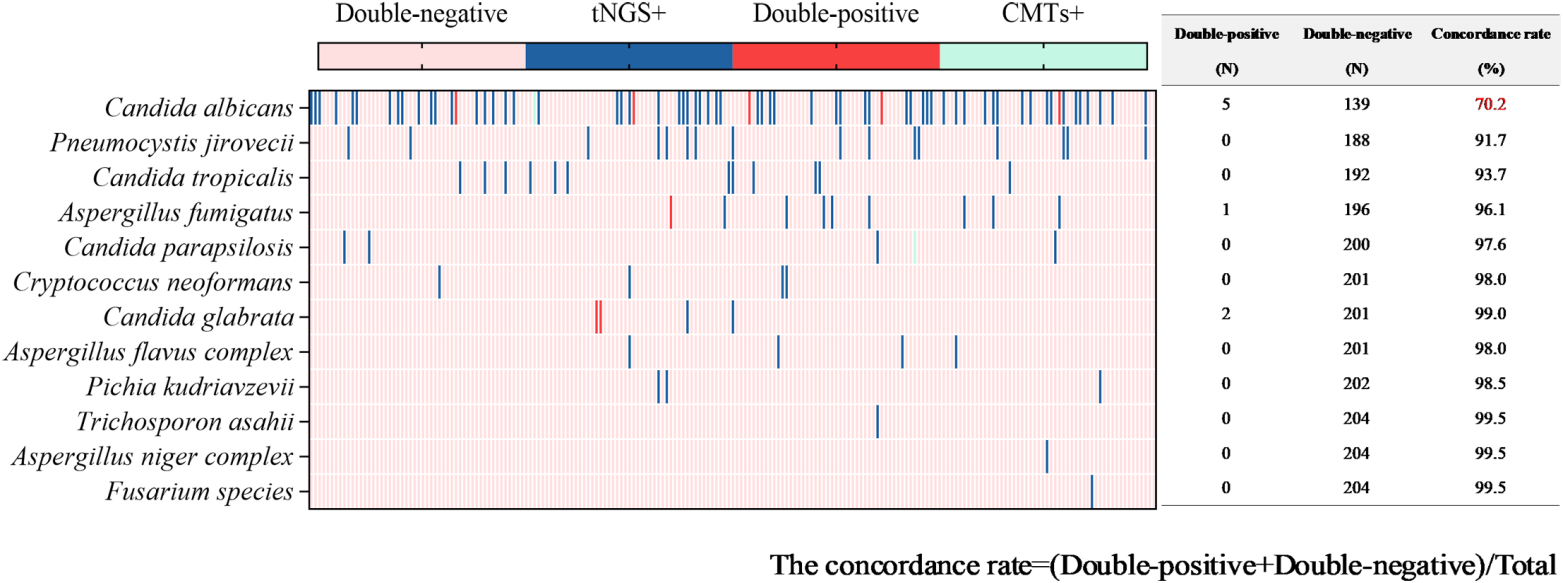
Concordance analysis between tNGS and CMTs assays in fungal pathogens. Heat map displaying the result of fungal pathogens detected by tNGS and CMTs.

Fungal detection was observed in 99 tests (48.3%) through tNGS, totaling 127 strains. This category mainly included *Candida albicans* (66 tests), *Pneumocystis jirovecii* (17 tests), *Candida tropicalis* (13 tests), and *Aspergillus fumigatus* (9 tests). Fungal pathogens were detected in 11 tests (5.4%) via CMTs (**Figure 4**). This category mainly included *Candida albicans* (5 tests) and *Candida glabrata* (2 tests). When using both methods to detect fungi, there were 11 double-positive tests, 105 double-negative tests, 89 tests where only tNGS was positive, and no test where only CMTs was positive. The concordance rate is shown in **Figure 6**. The concordance rate for *Candida albicans* is less than 90%, because the positive rate of tNGS is higher than that of CMTs. Compared to the reference standard of fungi culture and sputum smear, tNGS showed 100% sensitivity and 54.12% specificity (**Supplementary Figure 1B**). Among these, one case with completely inconsistent fungi detection between tNGS and culture was excluded from the statistical analysis.

### 3.5 Atypical pathogens identification by tNGS and CMTs

**Figure 7:**
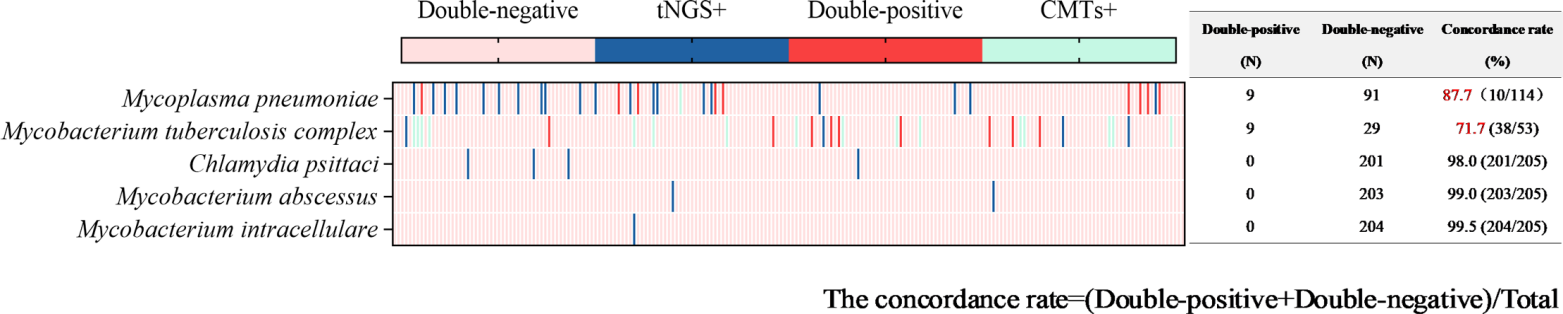
Concordance analysis between tNGS and CMTs assays in atypical pathogens. Heat map displaying the result of atypical pathogens detected by tNGS and CMTs.

Atypical pathogens were identified in 48 tests (23.4%) by tNGS, totaling 49 strains. *Mycoplasma pneumoniae* was the most prevalent (29 tests), followed by *Mycobacterium tuberculosis complex* (13 tests) and *Chlamydia psittaci* (4 tests). Atypical pathogens were detected in 34 tests (16.6%) by CMTs (**Figure 4**), including 24 tests of *Mycobacterium tuberculosis* (22 tests with T-SPOT.TB positive, 1 test with MTB PCR positive, and 1 test with sputum smear acid-fast staining positive), and 10 tests of *Mycoplasma pneumoniae* (4 seropositive for MP IgM and 6 positive for MP PCR). For the detection of atypical pathogens using both methods, 18 tests were positive by both, 31 tests were positive only by tNGS, 17 tests were positive only by the CMTs, and the concordance rate is shown in **Figure 7**. The concordance rate for *Mycoplasma pneumoniae* is less than 90%, because some *Mycoplasma pneumoniae* are tested only positive by tNGS while IgM antibody tests are negative. Based on comprehensive clinical information, 16 patients were diagnosed with *Mycobacterium tuberculosis* infection, among which 12 were positive by tNGS testing, 11 were positive by CMTs, and 9 were positive by both. The detection rates of *Mycobacterium tuberculosis* for tNGS and CMTs were 75.0% (12/16) and 68.8% (11/16), respectively. There was no statistical difference between the two (*P* > 0.05). However, the concordance rate for *Mycobacterium tuberculosis* is only 71.7%, which is attributed to the high false positive rate of the T-SPOT.TB test. As shown in **Supplementary Figure 1C**, the sensitivity and specificity of tNGS were 75% and 99.47%, respectively.

### 3.6 Virus pathogens identification by tNGS and CMTs

**Figure 8:**
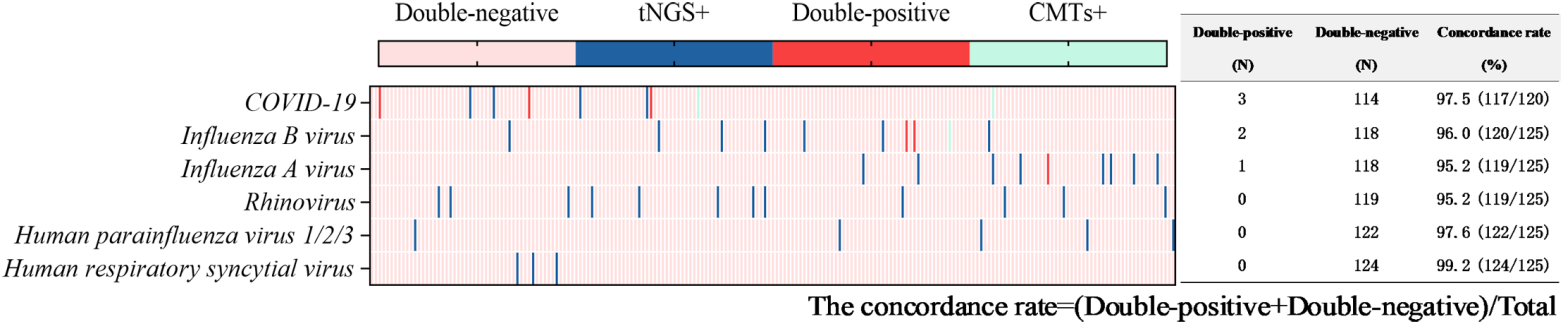
Concordance analysis between tNGS and CMTs assays in virus pathogens. Heat map displaying the result of virus pathogens detected by tNGS and CMTs.

Viruses were detected in 116 tests (56.6%) by tNGS, totaling 160 strains. The predominant virus detected was *Epstein-Barr virus* (EBV) (51 tests), followed by *Herpes simplex virus type 1* (27 tests), *cytomegalovirus* (13 tests), *rhinovirus* (12 tests), *influenza A virus* (9 tests), *influenza B virus* (9 tests), and COVID-19 (8 tests). Viruses were detected in 9 tests (4.4%) via CMTs (**Figure 4**), including 5 tests of COVID-19, 3 tests of *influenza B virus*, and 1 test of *influenza A virus*. Additionally, 154 tests (60.3%) tested positive solely via tNGS, and 3 tests was positive exclusively via CMTs. Only 6 tests were positive by both methods for virus detection. The concordance rates for viruses detectable by both methods are shown in **Figure 8**, with all concordance rates exceeding 90%. Then we further assessed the efficacy of tNGS in detecting COVID-19. Compared with the RT-qPCR results, tNGS exhibited 60% sensitivity and 99.13% specificity (**Supplementary Figure 1D**).

### 3.7 Clinical impact of tNGS

An analysis of the treatment records of 203 patients revealed that antibiotics were adjusted for 35 patients based on tNGS results, including 8 patients with bacterial infections (2 with *Klebsiella pneumoniae*, 2 with *Staphylococcus aureus*, 1 with *Fusobacterium nucleatum*, 1 with *Pseudomonas aeruginosa*, 1 with a mixed infection of *Klebsiella pneumoniae*, *Pseudomonas aeruginosa*, and *Acinetobacter baumannii*, and 1 with a mixed infection of *Klebsiella pneumoniae* and *Haemophilus influenzae*), 14 patients with fungal infections (9 with *Candida albicans*, 3 with *Pneumocystis jirovecii*, 1 with *Candida parapsilosis*, and 1 with *Aspergillus flavus complex*), 8 patients with atypical pathogen infections (2 with *Mycoplasma pneumoniae*, 4 with *Mycobacterium tuberculosis*, 1 with *Mycobacterium intracellulare*, and 1 with *Chlamydia psittaci*), 1 patient with a mixed bacterial and fungal infection (*Stenotrophomonas maltophilia* and *Fusarium* spp.), and 4 patients with viral infections (2 with *influenza B virus*, 1 with *human respiratory syncytial virus type 1*, and 1 with *Epstein-Barr virus*). Most of the unadjusted cases were related to empirical medications that had previously been effective in covering the detected pathogens. In addition, the average turnaround time (TAT) for tNGS is 1–2 days, significantly shorter than the 2–5 days required by CMTs. Compared with CMTs, tNGS provides rapid and consistent TAT.

### 3.8 Comparison between the drug resistance genes of tNGS and the drug sensitivity results of CMTs

The drug resistance genes detected by tNGS include those related to *Carbapenem-Resistant Organisms* (CRO), *Methicillin-Resistant Staphylococcus aureus* (MRSA), *Mycoplasma pneumoniae* resistance. The CRO-related resistance genes include Class A carbapenemase genes (blaKPC, blaSME, blaIMI, blaGES), Class B metallo-β-lactamase genes (blaNDM, blaIMP, blaVIM, blaSPM, blaGIM), and Class D carbapenemase genes (blaOXA-48). The MRSA-related resistance gene includes mecA. The mutation points in the 23S rRNA gene associated with *Mycoplasma pneumoniae* resistance include A2063G, A2064G, A2067G, and C2617G. In this study, 45 tests of drug resistance genes were detected, including 24 tests of the 23S rRNA A:2063G gene, 11 tests of the MecA gene, 8 tests of the blaNDM gene, and 2 tests of the blaKPC gene. *Klebsiella pneumoniae*, *Acinetobacter baumannii*, *Escherichia coli*, and *Pseudomonas aeruginosa* can harbor KPC and NDM resistance genes. Based on comprehensive clinical data, 57 tests were clinically diagnosed with infections caused by one or multiple pathogens, including *Pseudomonas aeruginosa*, *Klebsiella pneumoniae*, *Acinetobacter baumannii*, and *Escherichia coli*. Among these, 15 tests showed carbapenem resistance in the drug sensitivity tests. tNGS detected CRO resistance genes in 10 tests, and the resistance genotypes were consistent with the drug sensitivity results in 6 tests (7 times: the drug susceptibility report showed resistance to two types of bacteria in one test) (40%). tNGS detected 23 tests of *Staphylococcus aureus*, among which 11 tests harbored the mecA resistance gene. 6 tests were found to be positive for *Staphylococcus aureus* through culture, among which five tests showed resistance to methicillin-resistant *Staphylococcus aureus* (MRSA) based on drug sensitivity testing. Four out of these five tests (80%) were detected with the mecA resistance gene by tNGS (**Table 2**).

**Table 2:** Comparison between the drug resistance genes of tNGS and the drug sensitivity results of CMTs.

Pathogens	tNGS+ (N)	Culture+ (N)	Drug sensitivity+ (N)	Drug resistance genes (N)	Concordance (N,%)
*Klebsiella pneumoniae*	43	12	4	blaNDM 6 blaKPC 2	2, 13.3%
*Pseudomonas aeruginosa*	29	11	4	blaNDM 1 blaKPC 2	2, 13.3%
*Acinetobacter baumannii*	29	7	5	blaNDM 6 blaKPC 2	2, 13.3%
*Escherichia coli*	14	4	3	blaNDM 3 blaKPC 1	1, 6.7%
*Staphylococcus aureus*	23	6	5	mecA 11	4, 80%
*Mycoplasma pneumoniae*	29	10	NA	23SrRNA:A2063G 24	NA

The calculation of CRO concordance is based on 15 tests of drug-resistant bacterial infections confirmed by clinical drug sensitivity testing.

### 3.9 Monitoring of pathogen infection treatment status by tNGS

Two patients with complex infections were tracked to evaluate the effectiveness of tNGS in monitoring the treatment of critically ill patients and predicting drug resistance. The tNGS results were compared with the culture results throughout the treatment process, considering imaging findings and the antimicrobial agents administered for infection management.

**Figure 9:**
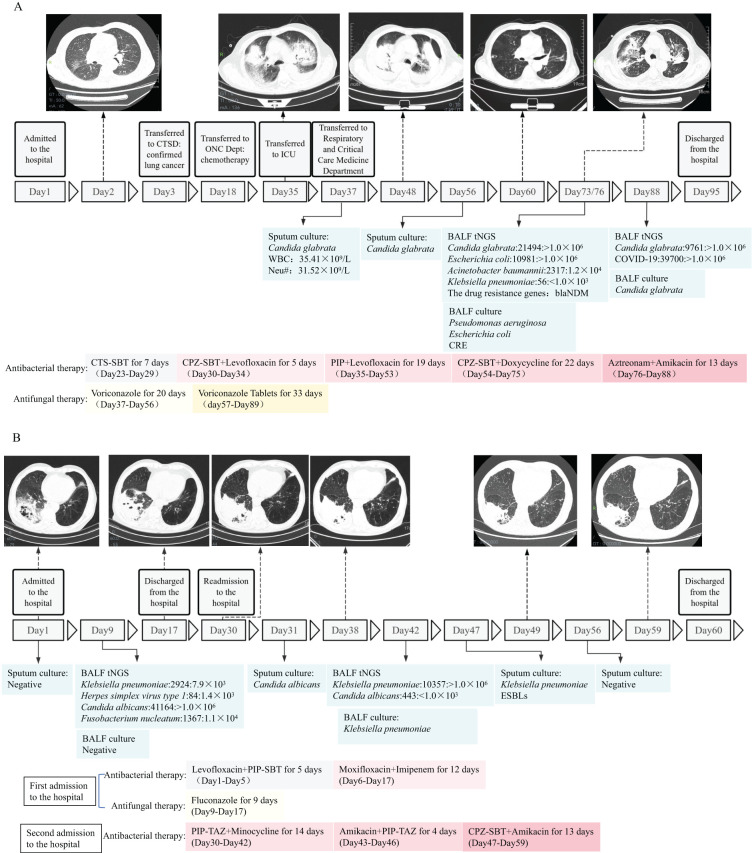
Schematic timeline of pathogen detection and treatment process of two patients with complicated infections (Case 1 and Case 2). **(A, B)** Profiles of microbiologic and imaging changes during anti-infective therapy in two patients who improved with treatment. CTS-SBT, Cefotaxime sodium and sulbactam sodium; CPZ-SBT, Cefoperazone sodium and sulbactam sodium; PIP, Piperacillin sodium; PIP-SBT, Piperacillin sodium and sulbactam sodium; PIP-TAZ, Piperacillin sodium and Tazobactam Sodium; CRE, Carbapenem-resistant Enterobacteriaceae; ESBLs, Extended-Spectrum Beta-Lactamase; CTSD, Cardiothoracic Surgery Department; ONC Dept, Oncology Deptartment.

Case 1: A male patient, 82 years old, was admitted to the hospital due to “headache and decreased hearing for one month” and was diagnosed with lung cancer. During chemotherapy, the patient experienced recurrent fever. On day 37 of admission, the sputum culture results showed: *Candida glabrata*, and blood routine test reported: WBC (total white blood cell count) 35.41×10^9^/L and Neu# (neutrophil count) 31.52×10^9^/L. Considering the patient’s concurrent fungal and Gram-positive cocci infection, anti-infective treatment (voriconazole, levofloxacin, piperacillin sodium and tazobactam sodium) were administered. After treatment, a follow-up CT scan showed no improvement in the inflammatory lesions, and infection indicators remained abnormal: PCT 4.56 ng/ml, CRP 123.25 mg/l, WBC 21.13×10^9^/L, Neutrophils 19.21×10^9^/L. On day 72 of admission, both culture and tNGS testing were performed on the BALF specimen. The tNGS results showed: *Candida glabrata*: 21494: >1.0×10^6^, *Acinetobacter baumannii*: 2317: 1.2×10^3^, and resistance gene blaNDM was detected. Culture results: *Candida glabrata*, *Pseudomonas aeruginosa*, *Escherichia coli*. Resistance mechanism: Carbapenem-resistant Enterobacteriaceae (CRE). The pathogens detected by both methods were consistent, as well as the resistance genes and drug sensitivity results. Based on the drug sensitivity results, aztreonam and amikacin were administered for treatment. On day 88 of admission, WBC, PCT, and CRP levels decreased compared to previous values, and another BALF specimen was re-collected for culture and tNGS testing. The tNGS results: *Candida glabrata*: 9761: >1.0×10^6^, and no resistance genes detected. The culture results: *Candida glabrata*. Neither culture nor tNGS detected any bacteria, so aztreonam and amikacin were discontinued. The patient was discharged on day 95 (**Figure 9A**).

Case 2: A 74-year-old male patient was admitted to the hospital due to “fever for 5 days”. Based on CT scans and inflammatory indicators, a pulmonary infection was confirmed, and empirical treatment (levofloxacin, piperacillin sodium and sulbactam sodium) was initiated. But the patient continued to experience recurrent fever, the antibiotics were adjusted to a combination therapy (moxifloxacin, imipenem and cilastatin sodium). On day 7 of admission, BALF was collected for tNGS and microbial culture. The tNGS results of BALF came back: *Klebsiella pneumoniae*: 2924: 7.9×10^3^; *Herpes simplex virus type 1*:84:1.4×10^3^; *Candida albicans*: 41164: >1.0×10^4^; *Fusobacterium nucleatum*:1367:1.1×10^4^. The culture results of BALF reported: no pathogenic bacteria detected. Based on the comprehensive etiological results, fluconazole was added to the treatment. A follow-up CT scan showed a reduction in inflammatory lesions, indicating effective treatment. However, the full course of anti-infective therapy had not been completed, the patient requested discharge and was processed accordingly. On day 30, the patient was readmitted to the hospital. CT results showed a slight increase in left lung lesions compared to before. Gram-negative rods and Gram-positive cocci were found in the sputum smear, and the sputum culture revealed *Candida albicans*, and antimicrobial treatment was administered. On day 40, BALF was collected again for tNGS and culture. The results of BALF tNGS: *Klebsiella pneumoniae*: 10357: >1.0×10^3^; the culture results also reported *Klebsiella pneumoniae*. According to the above results, *Klebsiella pneumoniae* was determined to be the primary pathogen. On day 47, a follow-up sputum culture was performed, and the results showed *Klebsiella pneumoniae* with a resistance mechanism of Extended-Spectrum Beta-Lactamase (ESBLs) bacteria. Based on the drug sensitivity results, the antimicrobial treatment was switched to a combination (amikacin, cefoperazone sodium and sulbactam sodium). CT scans on days 49 and 59 showed gradual improvement in bilateral lung lesions, and the patient was discharged on day 59 (**Figure 9B**).

## 4 Discussion

The tNGS method used in our study is a targeted detection of 198 respiratory pathogens (**Supplementary Table S**). It establishes an ultra-multiplex PCR library construction system to target and amplify the enriched sequences of interest, and applies next-generation sequencing technology for detection, achieving broad-spectrum and precise pathogen detection that can cover more than 95% of pathogens in the lower respiratory tract. Meanwhile, tNGS can detect resistance genes, including related genes for CRO, MRSA, and Mycoplasma pneumoniae resistance. Compared with mNGS, tNGS improves detection sensitivity, reduces costs, eliminates the influence of host sequences, and enables simultaneous detection of DNA and RNA, which is more advantageous for rapidly identifying target pathogens and precisely classifying them in clinical settings. A study by Sun W et al. demonstrated that the performance of mNGS and tNGS was comparable in detecting bacteria and fungi, when BALF samples from 83 patients suspected of having adult pulmonary infections were simultaneously tested using mNGS and tNGS, followed by PCR verification. However, tNGS may outperform mNGS in detecting DNA viruses, and there were no significant differences between the two NGS methods and PCR (Sun et al., 2024). Therefore, tNGS has started to be increasingly applied in clinical settings, addressing some of the challenges in clinical pathogen detection. However, the performance of tNGS in pathogen identification from BALF samples still requires further exploration and analysis, and there is a lack of large-scale comparative validation with CMTs.

This study aims to retrospectively analyze the results of tNGS and CMTs in 203 patients with confirmed pulmonary infections, and explore the efficacy of tNGS in etiological detection, so that it can truly exert its advantages of precision and speed in clinical practice. In the enrolled tests, tNGS identified more potential pathogens (56 vs. 20), including bacteria, viruses, fungi, and atypical pathogens. The positive rate of tNGS was higher than that of CMTs (99.5% vs. 35.6%), indicating that conventional methods failed to identify causative pathogens in many patients. Among the 73 double-positive patients, 91.8% of the patients showed complete or partial consistency between the two methods, indicating a high concordance rate between tNGS and CMTs. This study compared the consistency between the two methods and found that the consistency for common pathogens such as *Klebsiella pneumoniae*, *Fusobacterium nucleatum*, *Acinetobacter baumannii*, *Pseudomonas aeruginosa*, *Candida albicans*, and *Mycoplasma pneumoniae* was below 90%. This is due to the high sensitivity of tNGS technology, suggesting that it also has advantages in detecting common pathogens. Most specimens tested positive for two or more potential pathogens using tNGS, which can assist clinicians in the identification of mixed infections. The high sensitivity of tNGS may detect low-load colonization bacteria, and it is necessary to combine sequence count thresholds or microbial load ratios to distinguish infection from colonization. However, there is no unified standard for the sequence count threshold, the determination of colonizing bacteria must rely on the comprehensive analysis of the patient’s clinical manifestations, the effectiveness of antibiotic treatment, and professional knowledge (Society of Clinical Microbiology of China International Exchange and Promotion Association for Medical and Healthcare, 2024; The Laboratory Medicine Society, China Association of Medical Equipment, 2025). tNGS provides clinicians with a more comprehensive range of potential pathogens for disease analysis and diagnosis, thereby improving the efficiency of diagnosis and treatment. Additionally, this study intends to analyze antibiotic usage in patients and explore the impact of tNGS on clinical treatment. 35 patients (17.2%) had their antibiotic regimens modified based on tNGS results, achieving good therapeutic outcomes. Patients with pathogen-negative CMT results may derive greater diagnostic benefit from tNGS.

Two complex infection patients were tracked, among which one showed consistent pathogenic bacteria detected by both tNGS and culture before treatment, with consistent resistance genes and drug sensitivity results, and no bacteria were detected by either method after treatment. In the other patient, the first BLAF culture was negative, and based on comprehensive clinical data, it was considered that *Klebsiella pneumoniae* and *Candida albicans* were missed. After treatment, a follow-up BLAF tNGS and culture were performed, and both detected the same bacteria. This indicates that tNGS has a monitoring and evaluation role in the treatment of patients with pathogenic infections and serves as a complementary method to culture.

The main bacteria detected by tNGS include *Klebsiella pneumoniae* (43 tests), *Fusobacterium nucleatum* (43 tests), *Pseudomonas aeruginosa* (29 tests), *Acinetobacter baumannii* (29 tests each), *Staphylococcus aureus* (23 tests), *Haemophilus influenzae* (20 tests), *Streptococcus anginosus group* (19 tests), *Streptococcus pneumoniae* (16 tests), and *Escherichia coli* (4 tests). The main bacteria detected by CMTs contained *Klebsiella pneumoniae* (12 tests), *Pseudomonas aeruginosa* (11 tests), *Acinetobacter baumannii* (7 tests), *Staphylococcus aureus* (6 tests), *Escherichia coli* (4 tests), and *Haemophilus influenzae* (2 tests). The distribution of bacteria detected by both methods is roughly the same. However, tNGS detected 43 tests of *Fusobacterium nucleatum*, which was the bacterium with the highest positive rate, while CMTs did not detect any. This is because *Fusobacterium nucleatum* is a Gram-negative obligate anaerobic bacillus, and the environment for plate culture *in vitro* is difficult to meet its growth requirements. Due to the limited sensitivity and specificity of traditional culture-based pathogenic diagnostic methods, the prevalence of infections caused by anaerobes such as *Fusobacterium nucleatum* may have been underestimated before the advent of new laboratory detection technologies, such as molecular testing. tNGS detected 19 tests of *Streptococcus anginosus group*, while traditional culture did not. It is considered that the *Streptococcus anginosus group* is likely to be colonizing bacteria, thus they were excluded during the culture process. 16 tests of *Streptococcus pneumoniae* were identified by tNGS solely, presumably due to its high cultivation requirements and susceptibility to interference from other bacteria. This is consistent with the study by Chao L et al., which found that NGS has higher sensitivity for *Streptococcus pneumoniae* compared to culture, suggesting that NGS is a complementary approach for bacteria with low positive rates by culture (Chao et al., 2020).

The positive rate for fungi by tNGS significantly surpassed that of culture (48.3% vs. 5.4%). *Pneumocystis jirovecii* is a common opportunistic pathogenic fungus that often causes disease in immunocompromised individuals such as those with HIV infection, organ transplantation, hematological malignancies, and those using immunosuppressants (Bateman et al., 2020). Currently, *Pneumocystis jirovecii* cannot be cultured *in vitro*, and recent literature reports have suggested that mNGS can be used for the diagnosis of *Pneumocystis pneumonia* (Zhang et al., 2021; Li et al., 2023; Liu et al., 2023). In this study, tNGS detected 17 tests of *Pneumocystis jirovecii*, also revealing its potential use in the assessment of *Pneumocystis jirovecii* infections. tNGS detected 4 tests of *Cryptococcus neoformans*, 4 tests of *Candida glabrata*, 4 tests of *Aspergillus flavus complex*, 3 tests of *Candida krusei*, 1 test of *Trichosporon asahii*, 1 test of *Aspergillus niger complex*, and 1 test of *Fusarium* spp., all of which were not detected by culture, indicating the advantage of tNGS in the detection of rare fungal pathogens.

tNGS detected five atypical pathogens, constituted of *Mycoplasma pneumoniae* (29 tests), *Mycobacterium tuberculosis complex* (13 tests), *Chlamydia psittaci* (4 tests), *Mycobacterium abscessus* (2 tests), and *Mycobacterium intracellulare* (1 test), while CMTs only identified *Mycobacterium tuberculosis* and *Mycoplasma pneumoniae*, demonstrating that tNGS has advantages in detecting atypical pathogens, consistent with the study by Zhang P et al (Zhang et al., 2024). The *Mycoplasma pneumoniae* positive rates for tNGS and CMTs were 14.1% (29/205) and 8.77% (10/114), respectively. Resistance genes were detected by tNGS in 24 patients, with a resistance gene mutation rate of 82.8% (24/29). All resistance mutations were located at the 23S rRNA A:2063G gene locus, which can lead to a decrease in the affinity between macrolide molecules and ribosomes, thereby conferring resistance to macrolide antibiotics. Chen Y et al. reported that resistance gene analysis was conducted on 168 samples testing positive for *Mycoplasma pneumoniae*, and the results showed a resistance gene mutation rate of 88.10% (n=148). The most common resistance mutation site of our study was 23S rRNA A:2063G, which is consistent with the findings of another study (Chen et al., 2024). With the lifting of COVID-19 restrictions, *Mycoplasma pneumoniae* and genotypes resistant to macrolide antibiotics are showing a spreading trend in China (Yan et al., 2024; Wang et al., 2023). However, PCR and serological tests for *Mycoplasma pneumoniae* cannot assess drug susceptibility, limiting their clinical utility. tNGS can serve as a powerful tool in the epidemiological surveillance and diagnosis of *Mycoplasma pneumoniae*. There is no statistically significant difference in the detection rate of *Mycobacterium tuberculosis* between tNGS and CMTs (75.0% vs. 68.8%), demonstrating that both methods have comparable detection capabilities for *Mycobacterium tuberculosis*.

The virus positive rate of tNGS significantly surpassed that of CMTs (56.6% vs. 4.4%). Deng Z et al. reported that viruses were observed in 176 (84.2%) sputum samples by tNGS, with the main being *rhinovirus*, *cytomegalovirus*, *human herpesvirus 7*, and *Epstein-Barr virus*, which are roughly consistent with the main types of viruses detected in this study. However, the overall positive rate and the ranking of viruses differ, possibly due to the following reasons: (1) the types of specimens are different, as this study only collected BALF specimens; (2) the age of the study participants is different, with 71.3% of the participants in the study by Deng Z et al. being under 18 years old (Deng et al., 2023). CMTs lack the detection of *Epstein-Barr virus*, *human herpesvirus type 1*, and *cytomegalovirus*. Although these three viruses are not the main pathogens of acute respiratory viral infections, they often co-infect with other pathogens and may affect the prognosis of patients. Therefore, they still need to be given attention in clinical diagnosis and treatment (Zhang et al., 2024; Liu et al., 2022; Naigeon et al., 2023; Walton et al., 2014).

Another objective of our study is to explore the predictive capability of tNGS for drug-resistant bacteria by comparing the resistance genes detected with clinical drug sensitivity results. tNGS detected 10 tests of CRO resistance genes and 11 tests of mecA resistance genes, with 6 and 4 tests respectively showing consistency with clinical drug sensitivity results. There were still 6 tests where clinical drug sensitivity results displayed carbapenem resistance and 1 test indicating MRSA resistance, but tNGS did not detect any resistance genes. Although tNGS can provide clinicians with timely information for appropriate selection of antimicrobial agents, it still cannot replace the results of clinical drug sensitivity testing. There are various reasons leading to the inconsistency between resistant genotype and phenotype, such as the gene being located on a plasmid but not expressed, or the presence of other undetected resistance mechanisms (e.g., activation of efflux pumps). The study by Serpa PH et al. also shows that mNGS serves as a complement rather than a replacement for clinical drug sensitivity testing (Serpa et al., 2022). Additionally, when multiple bacteria were exhibited by tNGS, it is unable to distinguish which specific bacterium is resistant. In this study, among the 10 tests of CRO resistance genes, tNGS identified only one suspected associated bacterium in 3 tests, two suspected associated bacteria in 2 tests, three suspected associated bacteria in 4 tests, and four suspected associated bacteria in 1 test.

This study has certain limitations. Firstly, due to the lack of final pathogen diagnosis results in some cases, it was not possible to calculate the sensitivity and specificity of tNGS using clinical diagnosis as a gold standard, which hindered a comprehensive evaluation of its diagnostic performance. Secondly, the duration of this study was relatively short, and the sample size was limited, which may affect the stability of the study results.

## 5 Conclusion

Our research emphasizes the clinical application of pathogen diagnosis and drug resistance prediction based on BALF in patients with pulmonary infections. The results indicate that, compared with CMTs, tNGS can simultaneously detect bacteria, viruses, fungi, and atypical pathogens without prior knowledge of the infection source, and its positive rate is significantly higher, providing a more comprehensive view of the microbial community. This aids in the early identification of potential pathogens and improves diagnostic efficiency, especially for those highly pathogenic or rarely colonizing pathogens. This technology also supports clinical treatment decisions, treatment monitoring, and drug resistance prediction. In the future, prospective, multicenter, large-scale studies on the clinical application of tNGS in infectious diseases are needed, and comparisons of tNGS efficiency across different specimen types should be conducted.

## Data Availability

The original contributions presented in the study are included in the article/**Supplementary Material**. Further inquiries can be directed to the corresponding author/s.

## Supplementary material

Supplementary Figure 1 Contingency tables for the gold standard(Culture/PCR/Clinical Diagnosis)with tNGS. PPV, positive predictive value; NPV, negative predictive value.
