# Supplementary figures and images for: Application of targeted next-generation sequencing for pathogens diagnosis and drug resistance prediction in bronchoalveolar lavage fluid of pulmonary infections
Source: Front Cell Infect Microbiol. 2025 Jun 9;15:1590881. doi: 10.3389/fcimb.2025.1590881 (PMC12183283; doi:10.3389/fcimb.2025.1590881)

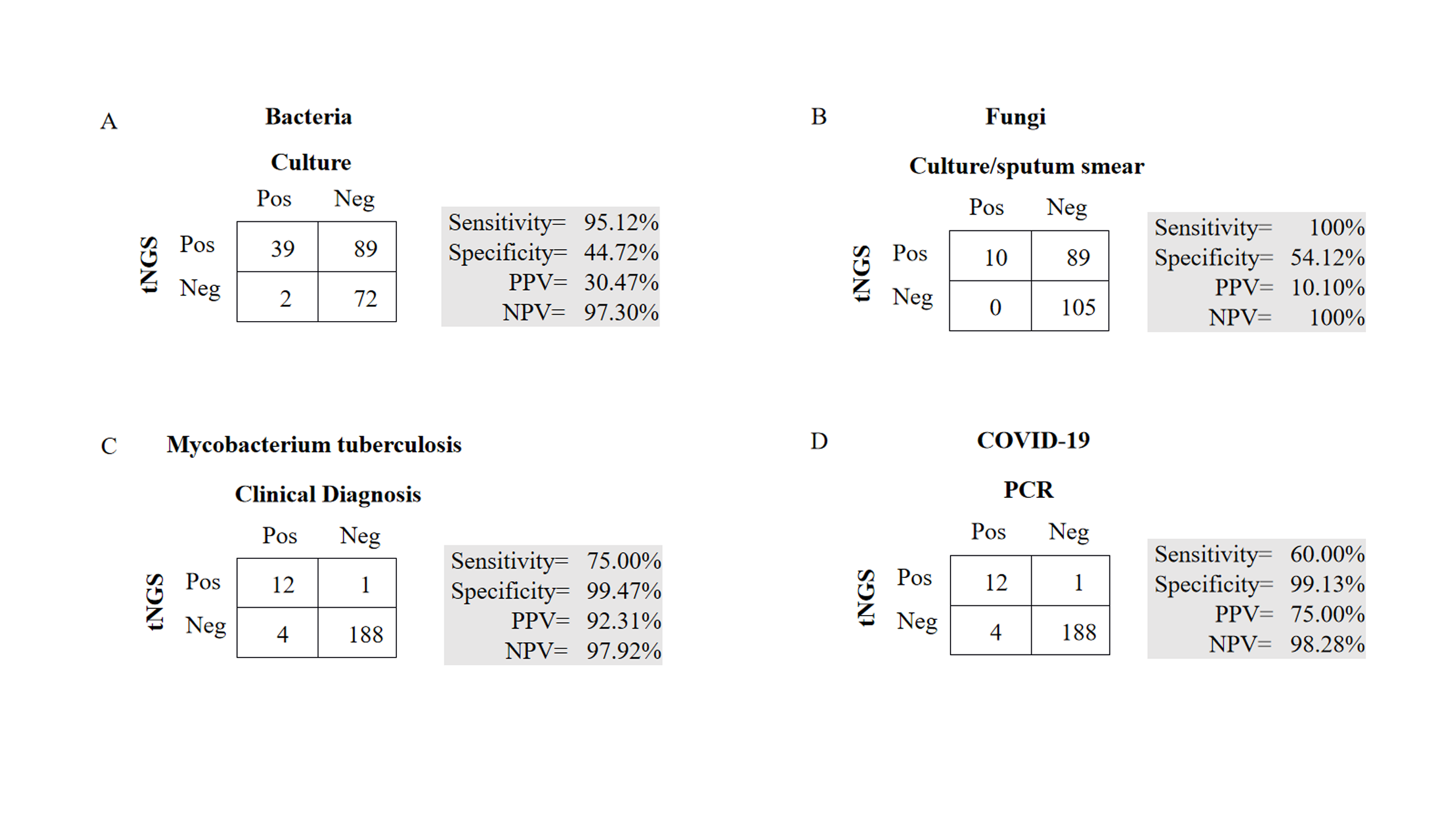

Supplement: Supplementary Figure 1 — Contingency tables for the gold standard(Culture/PCR/Clinical Diagnosis)with tNGS. PPV, positive predictive value; NPV, negative predictive value. [file Image1.tif]
